# Eye Movement Desensitization and Reprocessing and Slow Wave Sleep: A Putative Mechanism of Action

**DOI:** 10.3389/fpsyg.2017.01935

**Published:** 2017-11-07

**Authors:** Marco Pagani, Benedikt L. Amann, Ramon Landin-Romero, Sara Carletto

**Affiliations:** ^1^Institute of Cognitive Sciences and Technologies (CNR), Rome, Italy; ^2^Institut de Neuropsiquiatria i Addiccions, Centre Fòrum Research Unit, Parc de Salut Mar, Barcelona, Spain; ^3^Department of Psychiatry, Hospital del Mar Medical Research Institute, Autonomous University of Barcelona, Barcelona, Spain; ^4^Centro de Investigación Biomedica en Red de Salud Mental, Barcelona, Spain; ^5^Brain and Mind Centre and School of Psychology, The University of Sydney, Sydney, NSW, Australia; ^6^ARC Centre of Excellence in Cognition and its Disorders, Sydney, NSW, Australia; ^7^Department of Clinical and Biological Sciences, University of Turin, Turin, Italy

**Keywords:** EMDR, mechanism of action, eye movements, sleep, slow wave sleep, REM, orienting response, working memory

## Abstract

Eye Movement Desensitization and Reprocessing (EMDR) is considered highly efficacious for the treatment of Post-traumatic Stress Disorder and has proved to be a valid treatment approach with a wide range of applications. However, EMDR’s mechanisms of action is not yet fully understood. This is an active area of clinical and neurophysiological research, and several different hypotheses have been proposed. This paper discusses a conjecture which focuses on the similarity between the delta waves recorded by electroencephalography during Slow Wave Sleep (SWS) and those registered upon typical EMDR bilateral stimulation (eye movements or alternate tapping) during recurrent distressing memories of an emotionally traumatic event. SWS appears to have a key role in memory consolidation and in the reorganization of distant functional networks, as well as Eye Movements seem to reduce traumatic episodic memory and favor the reconsolidation of new associated information. The SWS hypothesis may put forward an explanation of how EMDR works, and is discussed also in light of other theories and neurobiological findings.

## Introduction

Eye Movement Desensitization and Reprocessing (EMDR) is a well-established psychological treatment for Post-traumatic Stress Disorder (PTSD) (Bradley et al., 2005; Chen et al., 2014). Furthermore, it has shown its efficacy in reducing anxiety levels in PTSD patients (Högberg et al., 2007, 2008; Bisson et al., 2013; Capezzani et al., 2013; McGuire et al., 2014; Faretta et al., 2016) and trauma-associated and psychiatric symptoms in various comorbid psychiatric diseases (Novo et al., 2014; Hase et al., 2015; Van Den Berg et al., 2015).

The neurobiological correlates of PTSD have been increasingly investigated by neuroimaging studies showing changes in cerebral blood flow (Bonne et al., 2003; Pagani et al., 2005; Lindauer et al., 2008; Nardo et al., 2011, 2015; for review see Bremner, 2007), metabolism (Pissiota et al., 2002; Osuch et al., 2008; Kim et al., 2012; Zhu et al., 2016), neuronal volume and density (Lindauer et al., 2004; Looi et al., 2009; Nardo et al., 2010, 2013; O’Doherty et al., 2015, 2017) and more recently in brain electric signal (Lee et al., 2014; Lobo et al., 2015), concordant with an involvement of the limbic system in the hyperarousal responsible for clinical symptoms. When reliving the traumatic events, the reduced control of the prefrontal cortex over hyperreactive amygdala and hippocampus is thought to be the core functional mechanisms of PTSD (Shin et al., 2006; Etkin and Wager, 2007).

Several neuroimaging investigations have demonstrated the effect of EMDR on cortical and sub-cortical regions involved in PTSD, depicting a clear association between disappearance of symptoms and the normalization of brain changes (Lansing et al., 2005; Pagani et al., 2007, 2012, 2015; Nardo et al., 2010; Landin-Romero et al., 2013; Trentini et al., 2015; Laugharne et al., 2016; for review see Pagani et al., 2013). Whole session monitoring of cortical activations by EEG made EMDR the first psychotherapy in which neurobiological correlates have been depicted in real time (Pagani et al., 2011, 2012).

A strong demand for the need of knowing how EMDR works has followed and here we shortly describe some of the hypotheses.

The original theory of Adaptive Information Processing (AIP) proposed by Shapiro (2001) stated that humans have an innate information processing system that stores new experiences into existing memory networks in an adaptive state. Pathology arises when new information is inadequately processed and then stored in a maladaptive mode. When memories are adequately processed, symptoms can be eliminated and memories integrated.

The orienting and relaxation response (OR) hypothesis offers a theoretical framework which may support the explanation that bilateral stimulation produces relaxation. The OR is a natural attentional reflex that can occur with any novel environmental stimulus increasing readiness to respond to danger (Wilson et al., 1996; Barrowcliff et al., 2003, 2004). The initial freeze response is accompanied by changes in autonomic responses. In the absence of danger, it is rapidly replaced with a feeling of relaxation holding the potential to desensitize the traumatic memory, suppressing its associated disturbance. Eye movements (EMs) trigger an OR that can (i) facilitate access to the traumatic memory without avoidance and (ii) cause subsequent rapid extinction after the determination of no immediate threat (Armstrong and Vaughan, 1996).

The working memory account postulates that a central executive system is responsible for the integration of information stored in different slave subsystems, i.e., the visuospatial sketchpad processing visual and spatial information (Baddeley and Hitch, 1974; Hornsveld et al., 2010, 2011; van den Hout et al., 2010, 2011, 2012, 2013). The dual task (i.e., the EMs and the visual imagery) draws on the limited-capacity of the slave subsystems and on the central executive working memory resources. EMs, competing with and disrupting working memory resources, change the somatic perceptions, reduce vividness and decrease the emotionality of traumatic imagery.

The thalamic binding model (Bergmann, 2008) posits that bilateral stimulation facilitates the activation of the ventrolateral and central lateral thalamic nuclei *via* lateral cerebellum, facilitating the integration of somatosensory, memory, cognitive, emotional, and synchronized hemispheric functions that are disrupted in PTSD.

These studies assigned an important role to EMs, which seem to be not only the underpinning mechanism of EMDR complementing traumatic memory extinction, but also the factor accounting for a faster response to treatment compared to other psychotherapies (Nijdam et al., 2012).

**FIGURE 1:**
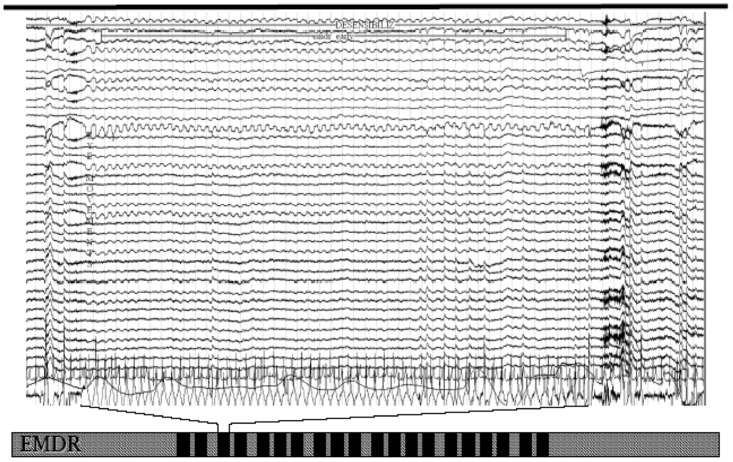
EEG tracing upon eye movements during an eye movement desensitization and reprocessing (EMDR) session. Note the slow wave sleep (SWS)-like frequency from the beginning to the end of bilateral stimulation.

It was recently highlighted (Pagani et al., 2012) that during successful EMDR therapy the cortical firing shifted from limbic structures toward regions with cognitive valence. In these studies, the occurrence of bilateral EMs was immediately accompanied by a synchronization of all cortical activity at a frequency in the delta range (**Figure 1**).

Despite these evidence, the role of EMs or, in general, bilateral stimulation in producing the neurobiological effects of EMDR is still unclear.

Based on this neurobiological evidence it is tempting to hypothesize a role of rapid eye movement (REM) and slow wave sleep (SWS) in the mechanism of action of EMDR. This follows the REM hypothesis for the mechanism of action of EMDR proposed by Stickgold (2002), according to which alternate bilateral stimulations typical of EMDR shift the brain into a memory processing mode similar to that of REM sleep.

Sleep has a bracing function, facilitates emotional processes and it is important for synaptic plasticity, emotional processing and memory formation. Long-lasting sleep disturbances are hallmark symptoms of PTSD that could interfere with a correct memory processing (Roszell et al., 1991; Leskin et al., 2002; Harvey et al., 2003) also causing functional and structural changes (Nardo et al., 2015).

## Physiological Basis of SWS and Memory Consolidation

To properly introduce our reasoning of a further role of SWS, it is essential to quote concepts and physiological bases well detailed in the works by Born et al. (2006) and Harper et al. (2009).

Memory recordings occurring during the waking state are temporarily stored in short-term memory and transferred to the neocortex during sleep. The combined episodic and emotional memory is replayed in the memory-editing matrix of the hippocampal-amygdalar complex as well as in neocortex during the first stage of SWS. In this process, memory is reinforced and extinguished by potentiation and depotentiation, respectively, of synapses of neurons recruited to form the memory chain. The excitatory glutamatergic pre-synaptic neurons release an amount of glutamate proportional to the strength of the signal. This in turn binds to the transmembrane α-amino-3-hydroxy-5-methyl-4-isoxazolepropionic acid glutamate receptor (AMPA), mediating the fast-synaptic transmission in central nervous system (CNS) networks, as the memory trace system. The opening of AMPA allows positively charged sodium into the post-synaptic neuron causing its depolarization. Stronger and repeated signals, as occurs during memory formation, cause more AMPA receptors to be transferred onto the surface of the post-synaptic neuron resulting in a larger sodium influx and in the opening of N-Methyl-D-aspartate (NMDA) glutamate receptors. This in turn favors the influx of positively charged calcium further strengthening the signal transmission. Synapses of the memory track are then potentiated, generating new proteins and gene expression resulting in the growth of new dendritic spines and new synaptic connections. The subsequent genetic expression and formation of permanent long-term memory occur mainly during REM sleep. In case of over-potentiation, low-frequency stimulation has a normalizing role depotentiating AMPA receptors, subsequently removed from the post-synaptic membrane, resulting in memory degradation. Following depotentiation, the receptors can no longer open and subsequently a substantial amount of them is removed from the post-synaptic membrane. The over-potentiated synapse is no longer able to convey the strong signals and henceforward is weakened. Potentiation and depotentiation (synaptic plasticity, adding or subtracting AMPA receptors) are carried out on synapses in the hippocampal-amygdalar complex and changes in their balance within the neural mechanisms of memory should be the molecular target for effective therapy.

Physiological normal sleep presents cyclic alternated pattern of REM and non-REM (SWS). EEG recordings show synchronous delta wave activity (0.5–4 cycles/s, i.e., 0.5–4 Hz) during SWS, and synchronous theta waves (4–8 Hertz) during REM sleep. SWS provides an optimal milieu for transferring edited memories from the hippocampus to the neocortex, as well as stimulating the integration of these into neocortical neuronal networks.

When new information is filtered by the sensorimotor cortex and simultaneously transferred to hippocampal networks, only the strong and repeated signals induce specific replication when the memory is replayed during SWS in the following night(s). During such phase, the cortical networks in which encoding originally took place produce slow oscillations (<1 Hz) that reactivate the hippocampal memory. This memory replay originates an input directed toward the same cortical synapses in synchronicity with high frequency activity originating in the thalamus. The combined action of these two signals, as described above, potentiates the synapses supporting the consolidation of long-term memory. In this phase, it is the combination and the alternation of slow and rapid waves that favors the transfer from hippocampus back to neocortex of the fresh memory encoded during the waking state. During REM sleep, also due to the absence of slow waves, there is a decrease of such activity suggesting a more intense memory consolidation at neocortical level. In this phase, new associations of emotional events mediated by limbic structures take place.

To summarize, during wakefulness autobiographical, emotional and potentially traumatic events are conveyed and represented into the sensorimotor cortex. From such perceptual representation system information are transferred to subcortical limbic structures as hippocampus (episodic) and amygdala (associated affect) where an initial formation and potentiation of memory occurs. During SWS global synaptic weakening along with slow consolidation of information take place. Relevant memory circuits are reactivated and long-term potentiation is induced. During REM sleep, a further potentiation of the reactivated connections in neocortical memory network occurs.

The recording of the episodic aspect of memory in the hippocampus results in a normal potentiation of hippocampal synapses. Traumatic events may cause over-potentiation of amygdalar synapses and all post-synaptic AMPA binding sites will be occupied by glutamate. In such circumstances, the transfer to neocortex mainly through anterior cingulate cortex cannot occur since memories need the same synchronized signal intensity at emotional and cognitive level for the correct processing. Fragmented non-processed episodic and traumatic memories are trapped in hippocampus or amygdala without the contextual integration needed to encode them in long-term memory in association neocortex and persist sometimes for life.

## The Link Between SWS and Bilateral Stimulation in EMDR

Bilateral stimulation typical of EMDR causes immediate slowing of the depolarization rate of neurons from the dominant waking state frequency of around 7 Hz to about 1.5 Hz (Harper et al., 2009; Pagani et al., 2011, 2012). The change of neuronal firing to low-frequency waves is a change from conditions favorable for synaptic potentiation to ones favorable for depotentiation.

In animals, low-frequency stimulation (5 Hertz) has shown to cause a depotentiation of amygdalar AMPA receptors involved in the retention of traumatic memory (Mao, 2006) and 900 stimuli at 1–5 Hz depotentiated synapses mediating memory (Kopp et al., 2006). This is about the number and the frequency of EMs during a typical EMDR session in which holding the attention on a traumatic memory targets the relevant synapses where it was originally encoded. It is worth noting that SWS occurs 3–5 times during night while bilateral stimulation is performed 25–30 times upon each EMDR session. This might account for the very fast processing of bad memories experienced by clients in a single or in a few EMDR sessions.

During EMDR sessions therapists performs bilateral stimulation at about 1–2 cycles/s (1–2 Hz) eliciting slow waves similar to the ones recorded during SWS. This suggests that memories aroused during therapy are continuously reactivated, replayed and encoded into existing memory networks.

A memory trace is weakened when held in attention and in such condition it is easily depotentiated. During an EMDR session the focus of the attention is on the fragmented traumatic memory and its synaptic traces in the amygdalar-hippocampal complex. EMDR decreases affective aspects of traumatic memories in the amygdala and leaves intact the associated cognitive aspects in the hippocampus. The affective and cognitive aspects of the memory are then merged in anterior cingulate cortex and sent to higher brain centers, where an encoding process within the association areas provide a clear distinction between the past and the present. The pathological memory trace is no longer confined by its over-potentiation to the limbic memory areas.

According to this model, desensitization indicated by the *D* in EMDR results from Depotentiation of fear memory synapses (Harper et al., 2009).

These speculations are supported by some recent neurophysiological findings. Harper et al. (2009) reported that, upon EMs, EEG tracing recorded in the delta range (1.5 Hertz) resembled the ones registered during SWS by Rétey et al. (2005). Such delta waves also paced β-waves (frequency of 13.5 Hertz), speaking in favor, during bilateral stimulation, of a general resonance in brain electric activity consonant with EMs. Recently, Pagani et al. (2011, 2012) in two separate investigations reported that the eye-movement component of EMDR induced an EEG pattern similar to the one described by Harper et al. (2009). This seems to confirm that the neurophysiological effect of bilateral stimulation by means of EMs or smooth pursuit (1–2 Hz) produces delta waves activity as during SWS (0.5–3 Hz).

It can be further speculated that the consolidation of emotional memory in neocortex during an EMDR session, often resulting in a sudden symptoms disappearance, is associated with periods in which slow (1.5 Hz) and fast (4–12 Hz, theta-alpha, typical waking state) waves are elicited by the alternation of bilateral stimulation and installation of positive cognition. This would mimic the previously described condition occurring during sleep in which memories are transferred from subcortical structures and encoded into neocortex.

If confirmed by future studies, the molecular and neurobiological mechanisms underlying our model could merge the effects explained by the OR theory, by the working memory account and by the hypothesis of Stickgold (2008).

In fact, we posit that bilateral stimulation mimics the low-frequency stimulation typical of SWS, inducing a depotentiation of the AMPA receptors of amygdalar synapses, which in turn lead to a weakening of the traumatic memory. This reduction of the over-potentiation of amygdalar synapses makes traumatic memory more accessible, and facilitates the connection between emotional memory and episodic memory, thus promoting a shift of memory to associative and neocortical areas. This is also consonant with the findings of Pagani et al. (2012) that showed in EMDR a shift of the traumatic memories from an implicit subcortical status to cortical regions that integrate them into existing semantic memory. Moreover, the depotentiation caused by low-frequency stimulation (i.e., EMDR bilateral stimulation) results in memory degradation and weakening, thus exerting the effect of reducing the vividness and emotionality of the traumatic memory, finally promoting a detachment from the past traumatic event.

These effects are the same described in clinical setting by the OR and the working memory models.

In assonance with OR hypothesis, delta waves elicited by bilateral stimulation facilitate the access to the dysfunctionally stored traumatic memory during wake consciousness. Thanks also to the absence of danger characterized by the therapeutic context, favoring relaxation, the extinction of traumatic memory and its reprocessing by associative and cortical areas could take place. The relaxation associated with the fading of the emotional memory is likely due to the reduction of the over-potentiation of amygdalar synapses occurring in real time during EMDR therapy.

Our speculation is also in agreement with the working memory account, since the effects of SWS-like neurophysiological conditions reproduced by EMDR bilateral stimulation, reducing in real time the over-potentiation of the amygdala and the relative hyperarousal, impact during therapy on vividness and on emotionality of traumatic memories, contributing to the sense of distancing from the original event described by patients.

Both models are based on the weakening of a memory when recalled and held in attention, but with different underlying explanations. In the working memory account, the imagery deflation effect is explained by the dual tasking (i.e., the competition between recall of the memory and the bilateral stimulation task) that affect the limited-capacity of the working memory. In our SWS model, memory degradation is determined by the depotentiation of AMPA receptors by EMDR bilateral stimulations miming SWS low-frequency stimulations occurring during sleep.

Lastly, our hypothesis follows the footsteps drawn by Stickgold (2002, 2008), deepening the role of SWS-like state induced by EMDR bilateral stimulation which promotes the transfer of episodic memory to semantic memory, that will be then consolidated during REM-like states.

Hippocampal-amygdala complex memories are transferred to neocortex, replayed, and consolidated into semantic associative memory networks. Information is then integrated to create meaning and learning from the event. The transfer might occur during slow-wave-sleep (1–3 Hz) and definitive memory consolidation during REM sleep (about 4–6 Hz). The traumatic episodic memory is weakened and then removed from hippocampus.

## Conclusion

In conclusion, this perspective article proposes that bilateral stimulation during EMDR might reproduce the neurophysiological conditions favorable for memory integration in associative neocortex, weakening the perception of the traumatic memory, reducing its vividness and inducing a sense of relaxation and safety.

Quoting Stickgold (2002): “*We are not claiming that we have solid evidence for all of the links and interpretations in the train of logic presented here.[…] Our goal is to demonstrate that there is a reasonable explanation of how EMDR works, which is consonant with modern neurobiology and cognitive neuroscience[…]*.*”*

Our aim is also to encourage further research in investigating the mechanisms of action of already proven effective psychotherapies such as EMDR, with experimental studies that might combine theoretical assumptions, molecular biology, neurophysiology, neuropsychology, brain imaging and clinical evidences in patients’ cohorts.

## Author Contributions

MP was responsible for the conception of the work, that was integrated and critically revised by SC, BA, and RL-R. All authors have approved the final manuscript.

## Conflict of Interest Statement

All authors have been invited as speakers in national and international EMDR conferences.
